# Whole-genome resequencing of the Kazakh coarse-wool fat-tailed sheep identifies functional variants in disease-associated genes

**DOI:** 10.3389/fvets.2026.1936482

**Published:** 2026-09-22

**Authors:** Darkhan Smagulov, Shynggys Orkara, Kisun Pokharel, Nurlan Sandybayev, Saltanat Khamzina, Primkul Ibragimov, Aigerim Khamzina

**Affiliations:** 1 West Kazakhstan Innovation and Technological University, Uralsk, Kazakhstan; 2 Kazakh National Agrarian Research University, Almaty, Kazakhstan; 3 Natural Resources Institute Finland (Luke), Jokioinen, Finland; 4 LLP Scientific and Educational Center “Qazyna”, Almaty, Kazakhstan

**Keywords:** disease resistance, indigenous breed, Kazakhstan, population structure, PRNP, sheep, TMEM154, whole-genome resequencing

## Abstract

The Kazakh coarse-wool fat-tailed sheep is an indigenous breed of Central Asia and represents a valuable genetic resource adapted to harsh continental environmental conditions. Despite its economic importance, its genomic architecture, population structure, and the genetic basis of disease resistance remain largely unexplored. Whole-genome sequencing was performed on 20 Kazakh coarse-wool fat-tailed sheep using the Illumina NovaSeq 6000 platform. Population structure was assessed by principal component analysis (PCA) using comparative genomic data from six domestic sheep breeds and the wild ancestor *Ovis orientalis*. Selection signatures were identified by integrating FST, nucleotide diversity (*π*), and Tajima’s *D* statistics. Variants within the *PRNP* and *TMEM154* loci were extracted from the filtered VCF files using bcftools. PCA revealed a distinct genetic cluster of the Kazakh coarse-wool fat-tailed sheep, showing close genetic affinity to Bashibai and Hu sheep while remaining clearly differentiated from European meat and short-tailed breeds. A total of 208 candidate selective sweep regions containing 297 overlapping genes were identified. Quantitative trait loci (QTL) annotation revealed enrichment for loci associated with resistance to gastrointestinal nematodes (14 QTLs), body weight (9 QTLs), susceptibility to pneumonia (2 QTLs), clinical mastitis (2 QTLs), and susceptibility to *Mycobacterium avium* subsp. *paratuberculosis* infection (2 QTLs). Five *PRNP* haplotypes were identified, with 60% of animals classified as high-risk (NSP3) for scrapie susceptibility. No animals carrying the fully resistant ARR/ARR genotype were detected. At the *TMEM154* locus, 95% of individuals carried the E35E genotype, which is associated with increased genetic susceptibility to Maedi-Visna. This study provides the first comprehensive genomic characterization of the Kazakh coarse-wool fat-tailed sheep, revealing its unique population structure, genomic regions under selection associated with adaptation and disease resistance, as well as genetic predisposition susceptibility-associated genotypes for scrapie and Maedi-Visna. These findings provide a valuable genomic resource for marker-assisted breeding, genetic improvement, and conservation of indigenous sheep genetic resources in Kazakhstan.

## 1 Introduction

Sheep represent one of the most economically important livestock species worldwide, making a substantial contribution to the production of meat, wool, and milk (1). Central Asia is home to unique indigenous sheep populations adapted to extreme continental climates and extensive grazing management systems (2, 3). Fat-tailed sheep constitute the foundation of the sheep industry in Kazakhstan, accounting for more than half of the country’s total sheep population, which reached 23.1 million head by 2025 (4, 5). The Kazakh coarse-wool fat-tailed (KCWFT) sheep is an indigenous breed that was formed under the arid and semi-arid ecosystems of Kazakhstan and is characterized by high endurance, the ability to efficiently utilize poor pastures, resistance to extreme seasonal temperature fluctuations, and a pronounced fat-tail type of fat deposition (2). Despite its economic and genetic importance, the genomic architecture, population structure, and genetic determinants of disease resistance in this breed remain insufficiently studied, and whole-genome sequencing (WGS) data for the KCWFT sheep are absent from public repositories (2, 4, 6).

WGS has revolutionized livestock genomics by enabling comprehensive characterization of genetic variation without the limitations of preselected markers (7, 8). Compared with SNP microarray genotyping, WGS reveals the complete spectrum of genetic diversity, including rare variants, structural variations, and population-specific polymorphisms (7, 9). In sheep, WGS has been widely applied to identify genomic signatures of selection associated with adaptation, production traits, and disease resistance. This approach has enabled the identification of numerous candidate genes associated with reproductive performance, wool characteristics, fat deposition, and the functioning of innate and adaptive immunity (9, 10).

For the functional interpretation of the identified genomic regions and candidate genes, they are widely compared with quantitative trait loci (QTLs) associated with economically important and adaptive traits (6, 7, 11, 12). In global sheep breeding, QTLs have been mapped for a wide range of production, adaptive, and immunological traits, including body weight, wool characteristics, resistance to gastrointestinal nematodes, and genetic susceptibility to pneumonia, clinical mastitis, and paratuberculosis (5, 13–15). However, QTL data for local Asian breeds remain extremely limited due to insufficient characterization of their genomes and the underrepresentation of these populations in international genetic databases, while the application of existing commercial SNP panels is complicated by the fact that they were developed primarily using European (4, 6, 12, 16).

Along with production traits, the study of genetic resistance to infectious diseases is an important direction in genomic selection, contributing to biosafety and the export potential of sheep breeding. Genetic predisposition of susceptibility of sheep to scrapie is determined by polymorphisms in the prion protein gene (*PRNP*), with codons 136, 154, and 171 being of key importance: the ARR haplotype is associated with resistance to classical scrapie, whereas VRQ is associated with high susceptibility (17–19). Similarly, genetic susceptibility to Maedi-Visna, caused by small ruminant lentiviruses (SRLV), is associated with polymorphism of the *TMEM154* gene, including the E35K mutation associated with increased resistance (20–22). Information on the epidemiological status of these diseases in Kazakhstan remains limited. Although no cases of scrapie or Maedi-Visna have been officially reported in Kazakhstan, this should not be interpreted as evidence of their absence. Monitoring of *PRNP* and *TMEM154* polymorphisms may nevertheless provide a useful complementary tool for genetic surveillance and breeding programs aimed at reducing genetic predisposition to these diseases in accordance with the veterinary and sanitary requirements of the Eurasian Economic Union (EAEU) and the global market (23–25).

Previous genomic studies of Kazakh sheep have mainly relied on medium-density SNP arrays and analyses of population structure, genetic diversity, runs of homozygosity (ROH), and genomic inbreeding. Although these studies have provided valuable insights into the genetic relationships and demographic history of local breeds, they were constrained by predefined marker sets and therefore could not comprehensively characterize rare and breed-specific variants, functional polymorphisms, structural variation, or disease-associated loci. Consequently, the genomic architecture and adaptive genetic variation of the KCWFT sheep remain largely unexplored.

Thus, the present study provides the first comprehensive whole-genome characterization of the KCWFT sheep breed using high-coverage WGS. Unlike previous studies based primarily on SNP arrays, this work enables genome-wide discovery of breed-specific genetic variation, characterization of selection signatures at nucleotide resolution, integration of these regions with economically important QTLs, and assessment of functional polymorphisms associated with hereditary disease resistance. These analyses provide new biological insights into the evolutionary history, adaptive potential, and veterinary genetic background of this indigenous breed. Therefore, the aim of this study was to perform a comprehensive genomic characterization using WGS, including analysis of population structure, identification of selection signatures and their associated QTLs, and evaluation of PRNP and TMEM154 gene polymorphisms associated with resistance to scrapie and Maedi-Visna (see Figure 1).

**Figure 1:**
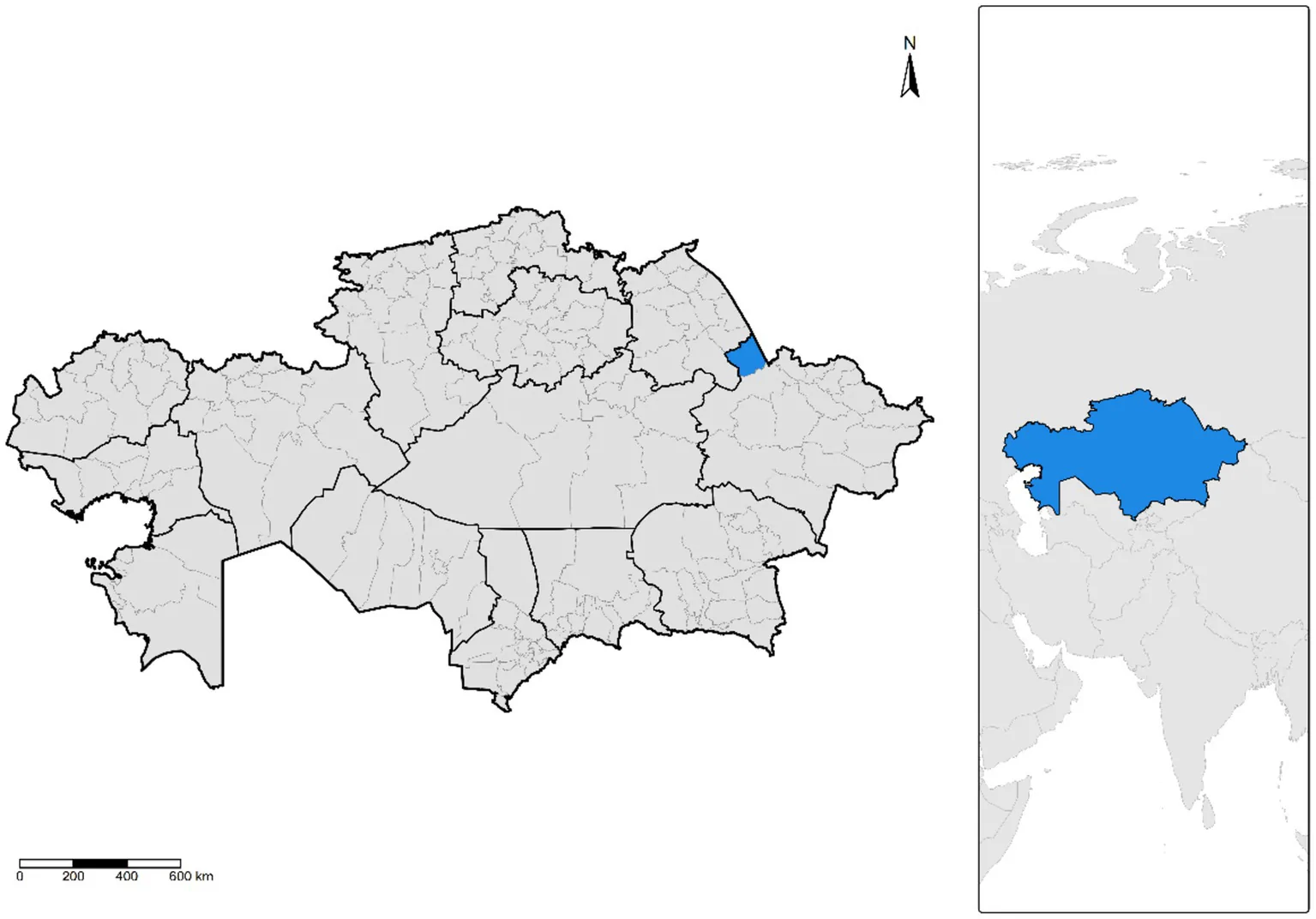
Sampling location map of KCWFT sheep (KCWFT). The Beskaragay district (Abai region) is highlighted in blue on the main map of Kazakhstan. Other districts are light gray. The inset shows Kazakhstan in dark blue within.

## 2 Materials and methods

*Institutional Review Board Statement*: The study was approved by the Bioethics Commission of PHPEI “West Kazakhstan Innovation and Technology University” Kazakhstan (approval date: 02 September 2024, protocol number 15/09).

### 2.1 Sampling, DNA extraction and sequencing

The study utilized ear tissue from 20 KCWFT sheep. The samples were collected from the Bepen farm in the Beskaragay District, Abay region, Republic of Kazakhstan (Figure 1). The ear notches were approximately 2-3 mm in diameter. Animals were selected to minimize close genetic relationships based on the available pedigree information. After collection, the samples were placed in sterile tubes and stored at −20 °C until DNA extraction. The KCWFT breed is traditionally raised in the eastern and central regions of Kazakhstan under extensive grazing conditions, and the Bepen farm represents one of its breeding populations.

Genomic DNA was extracted from ear tissue samples using the commercial PureLink™ Genomic DNA Mini Kit (Thermo Fisher Scientific, Waltham, MA, United States) according to the manufacturer’s protocol. DNA degradation and sample contamination were assessed by electrophoresis on a 1% agarose gel. DNA concentration and purity were determined using a NanoDrop 2000 spectrophotometer (Thermo Fisher Scientific, Wilmington, DE, United States) and a Qubit 3.0 fluorometer (Life Technologies, Carlsbad, CA, United States) with the dsDNA HS Assay Kit. WGS was performed on the Illumina NovaSeq 6000 platform (Illumina Inc., San Diego, CA, United States) using paired-end 150 bp reads (PE150) with single-index libraries, achieving 15X sequencing coverage per individual.

### 2.2 Quality control and reference alignment

Quality control of raw FASTQ files was performed using FastQC v0.12.1 (26), and the results were aggregated using MultiQC v1.30 (27). Adapter trimming and quality filtering were performed using fastp v0.24.0 (28).

Filtered reads were aligned to the *Ovis aries* ARS-UI_Ramb_v3.0 reference genome (assembly accession GCF_016772045.2) using the BWA-MEM v0.7.18 algorithm (29). Alignment files were converted to BAM format and sorted using SAMtools v1.3.1 (30). Duplicate reads were marked using samtools markdup and excluded from subsequent analyses.

### 2.3 Variant calling and annotation

Variant calling was performed using bcftools v1.16.1 (31), employing the mpileup utility with a minimum mapping quality of 20 (MAPQ ≥ 20) and a minimum base quality of 20 (BQ ≥ 20). SNPs and indels were called using bcftools call in multiallelic calling mode (−mv). Variants were called separately for each contig using bcftools mpileup, and subsequently merged into a single VCF file using bcftools concat. To obtain high-confidence variants, breed-specific VCF files were filtered using BCFtools v1.16.1 (31). Only variants with a call quality of at least 30 (QUAL ≥ 30), a total sequencing depth of at least 10× (INFO/DP ≥ 10), and a mean mapping quality of at least 40 (MQ ≥ 40) were retained. Filtered variants were indexed using bcftools index. Quality control at each stage was performed using bcftools stats, including assessment of the total number of SNPs and indels, variant quality distribution, sequencing depth, and the proportion of missing genotypes.

The initial dataset contained 42,278,564 variants (38,432,377 SNPs and 3,846,187 indels). After filtering, 39,864,460 high-quality variants remained (36,130,140 SNPs and 3,734,320 indels).

Functional annotation of the identified variants was performed using SnpEff 5.4c (32), with the annotation database for the ARS-UI_Ramb_v3.0 assembly. After annotation, only autosomal biallelic single nucleotide polymorphisms (SNPs) were retained for subsequent analyses using the command bcftools view -m2 -M2 -v snps, thereby excluding multiallelic variants and indels and generating a high-quality set of biallelic SNPs for downstream analyses of genetic diversity and association studies.

### 2.4 Population structure analysis

Prior to population structure analysis, variants were filtered to remove those with a call rate below 90% and a minor allele frequency (MAF) below 0.05. To reduce redundancy caused by linkage disequilibrium (LD), LD pruning was performed using PLINK v2.00a6.12. The filtering parameters were a sliding window of 50 SNPs, a step size of 5 SNPs, and an *r*^2^ threshold of 0.2 (--indep-pairwise 50 5 0.2). SNPs exceeding the specified LD threshold within each window were removed. Based on the filtered set of independent SNPs, the first 10 principal components were calculated using the --pca command in PLINK v2.00a6.12. Visualization was performed in R v4.5.2 using the ggplot2 v3.5.1 package.

For comparative analysis, raw FASTQ files from 72 animals representing six additional sheep breeds (NCBI accession: PRJNA624020), as well as ten samples of the wild ancestor *Ovis orientalis* (NCBI accession: PRJEB3139), were retrieved from the European Variation Archive (EVA) (Supplementary file 1). All comparative samples were processed using the same bioinformatics pipeline and aligned to the ARS-UI_Ramb_v3.0 reference genome.

Pairwise Fst was calculated using PLINK (option --fst), weighted Fst according to the method of Weir & Cockerham (1984). All calculations were performed based on 15,445,468 biallelic SNPs after QC filtering.

### 2.5 Selection signature analysis

Genomic regions under selection were identified using three metrics: Fst (population differentiation), nucleotide diversity (*π*), and Tajima’s *D* statistic. High Fst values indicate inter-population differentiation, reduced *π* indicates loss of genetic diversity within the population, and negative Tajima’s *D* values are characteristic of recent positive selection, and negative Tajima’s *D* values indicate an excess of low-frequency variants that may occur following a recent selective sweep. The combined use of these complementary statistics provides more robust evidence for candidate selection signatures than reliance on a single metric, as they capture different aspects of genomic variation. All three statistics were calculated in sliding windows using the parameters --fst-window-size 50,000 --fst-window-step 25,000 in VCFtools v0.1.16. Candidate regions under selection were defined as windows simultaneously falling within the upper 5% of the empirical Fst distribution (≥95th percentile), the lower 5% of the nucleotide diversity (*π*) distribution (≤5th percentile), and the lower 5% of the Tajima’s *D* distribution (≤5th percentile). Requiring concordant evidence from all three statistics was used to increase the robustness of candidate region identification and reduce reliance on signals derived from a single metric. Genes located within these regions were extracted using bedtools v2.31.1. Identified candidate genes were annotated using the Ensembl database (*Ovis aries* Rambouillet, release 115). The genomic coordinates of the annotated genes were subsequently compared with the QTLdb database (version 59, May 20, 2026).

### 2.6 Analysis of *PRNP* and *TMEM154* gene polymorphisms

Variants within the *PRNP* (NC_056066.1:46,638,372–46,658,965) and *TMEM154* (NC_056070.1:5,175,905–5,228,028) loci were extracted from the VCF file using bcftools view -r, while retaining the quality thresholds (QUAL ≥ 30, INFO/DP ≥ 10, MQ ≥ 40). PRNP polymorphisms were determined at codons 136 (position NC_056066.1:46,655,342; rs591379086), 154 (NC_056066.1:46,655,396; rs605048948), and 171 (NC_056066.1:46,655,447; rs160575103). Haplotypes were reconstructed by direct counting of allele combinations; in cases of heterozygosity at multiple codons, phasing was determined using overlapping sequencing reads. Risk classification according to the United Kingdom National Scrapie Plan (NSP) was performed using the standard classification scheme (33, 34).

The *TMEM154* gene polymorphism was analyzed at the E35K position (NC_056070.1:5200664, rs408593969). Genotypes were classified into three groups: resistant (KK35 homozygotes), intermediate (EK35 heterozygotes), and susceptible (EE35 homozygotes for the ancestral allele). Considering the dominant inheritance of susceptibility-associated alleles, carriers of at least one E35 allele (EK and EE genotypes) were considered to have increased genetic predisposition (20). Allele and genotype frequencies were calculated by direct counting.

## 3 Results

### 3.1 Sequencing, mapping, filtering, and annotation results

WGS generated 571 GB of data, with an average of 28.6 GB per sample. A total of 8.3 billion paired-end reads of 150 bp were obtained. The average mapping rate to the *Ovis aries* ARS-UI_Ramb_v3.0 reference genome was 99.70% (±0.31%), ranging from 98.43 to 99.87%. The proportion of properly paired reads averaged 98.31%. The mean sequencing depth was 14.6 × (range: 11.9×–21.2×) (Supplementary file 2).

The initial variant analysis identified 42,278,564 variants (38,432,377 SNPs and 3,846,187 indels). After quality filtering, 39,864,460 high-quality variants remained (36,130,140 SNPs and 3,734,320 indels). The transition-to-transversion (Ts/Tv) ratio was 2.45, indicating the high quality of the called variants (Figure 2).

**Figure 2:**
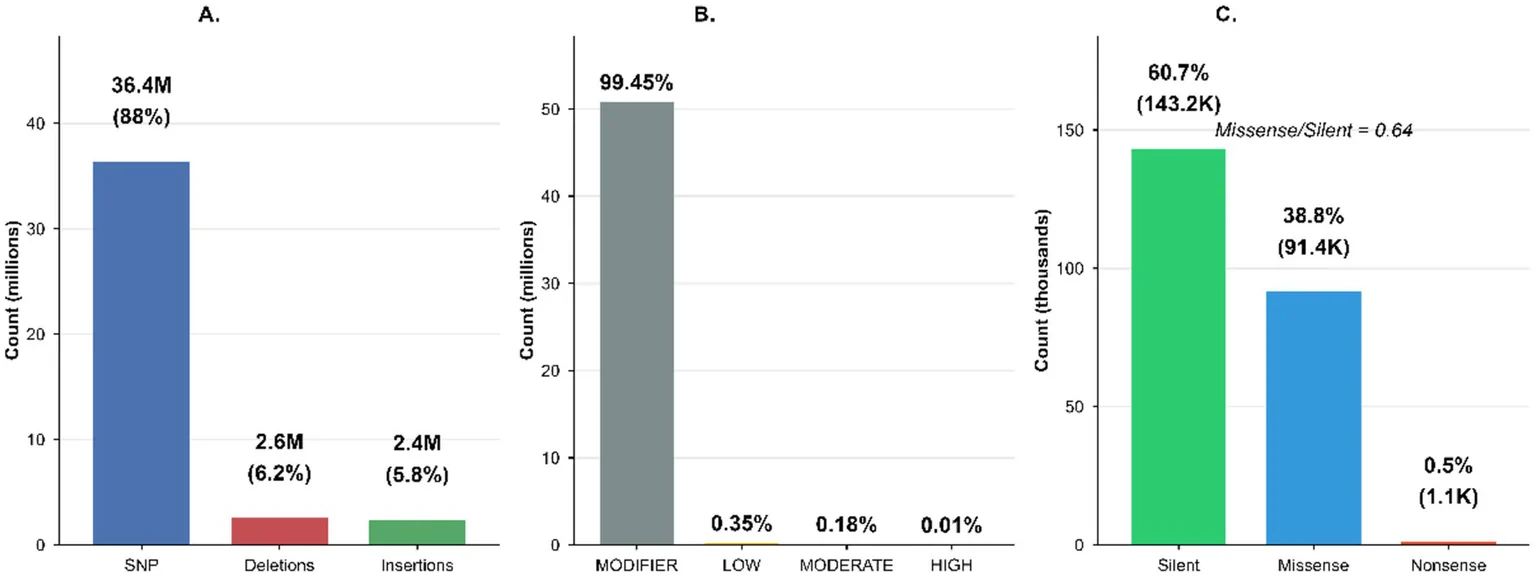
Functional annotation of identified variants in KCWFT WGS data. **(A)** Variant types (SNP, insertions, deletions). **(B)** Predicted impact categories (MODIFIER, LOW, MODERATE, HIGH). **(C)** Functional classes of coding variants (silent, missense, nonsense).

Functional annotation of the identified variants showed that the majority of polymorphisms were located in non-coding regions of the genome. The largest number of SNPs was detected in intergenic regions (20,618,819; 40.361%) and intronic regions (17,086,862; 33.447%). Among the functionally significant categories, missense variants (91,147; 0.178%) and synonymous substitutions (143,080; 0.28%) were identified. High-impact mutations included variants resulting in premature stop codons (stop_gained—1,179), loss of start codons (start_lost—182), or loss of stop codons (stop_lost—128). Variants potentially affecting splicing processes were also identified, including substitutions at splice donor sites (1,147) and splice acceptor sites (1,093), as well as in the adjacent splice regions (35,778) (Table 1). These results indicate the presence of a substantial number of functionally significant variants capable of modifying biological processes and determining the adaptive plasticity of this breed.

**Table 1:** Summary of functional annotation for identified variants in KCWFT.

Category	Count	Percentage (%)
Intergenic region	20,618,819	40.36
Intron variant	17,086,862	33.45
Downstream gene variant	4,116,028	8.06
Upstream gene variant	4,084,164	7.99
Intragenic variant	2,897,082	5.67
Non-coding transcript exon variant	1,774,054	3.47
Synonymous variant	143,080	0.28
Missense variant	91,147	0.18
3′ UTR variant	178,513	0.35
5′ UTR variant	46,213	0.09
Splice region variant	35,778	0.07
Frameshift variant	1,499	0.003
Stop gained	1,179	0.002
Splice acceptor/donor	2,240	0.004
Other	6,493	0.01

### 3.2 Population structure

Phylogenetic analysis (Figure 3) demonstrates a clear division of the sheep breeds studied into distinct clusters corresponding to their breed affiliation. All KCWFT samples form an independent, well-defined clade (red), indicating high genetic homogeneity within the breed and pronounced genetic differentiation relative to the other breeds studied.

**Figure 3:**
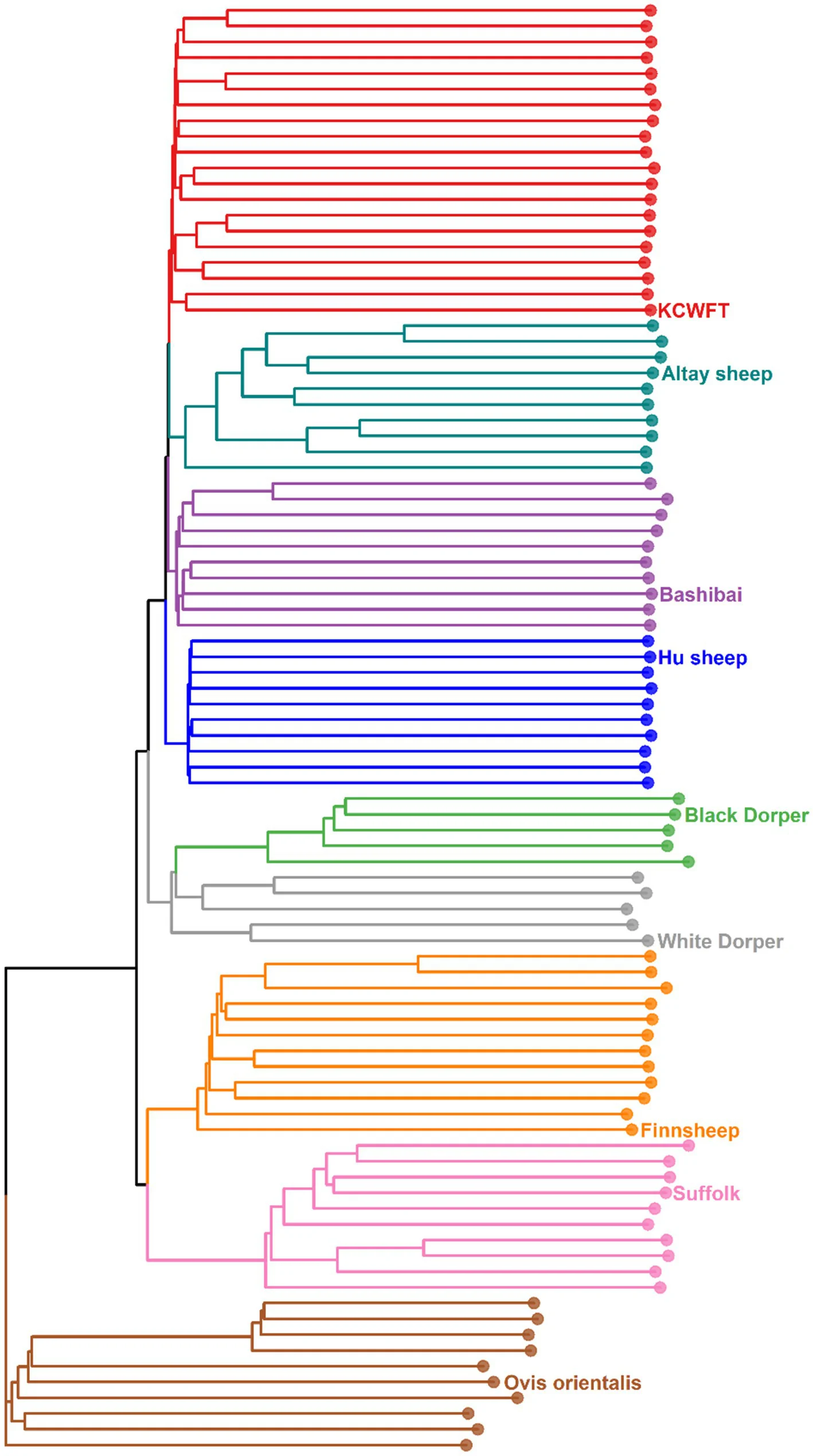
Phylogenetic relationships among the studied sheep populations based on whole-genome sequencing (WGS) data. The tree includes KCWFT sheep and the comparative Altay, Bashibai, Hu, Black Dorper, White Dorper, Finnsheep, and Suffolk breeds, with *Ovis orientalis* included as the wild reference population. Individuals are color-coded according to breed/population. The scale bar indicates genetic distance.

Principal component analysis (PCA) based on the set of LD-independent SNPs revealed clear genetic differentiation among the studied populations. The first principal component (PC1) separated the wild ancestors (*O. orientalis*) from all domestic breeds, reflecting a clear genetic separation associated with the domestication process, whereas the second principal component (PC2) reflected the geographical patterns of breed formation. As shown in Figure 4, the KCWFT sheep formed a single cluster, demonstrating close genetic affinity to the Asian fat-tailed breeds Bashibai (BSB), Hu (HU), and Altay (AL).

**Figure 4:**
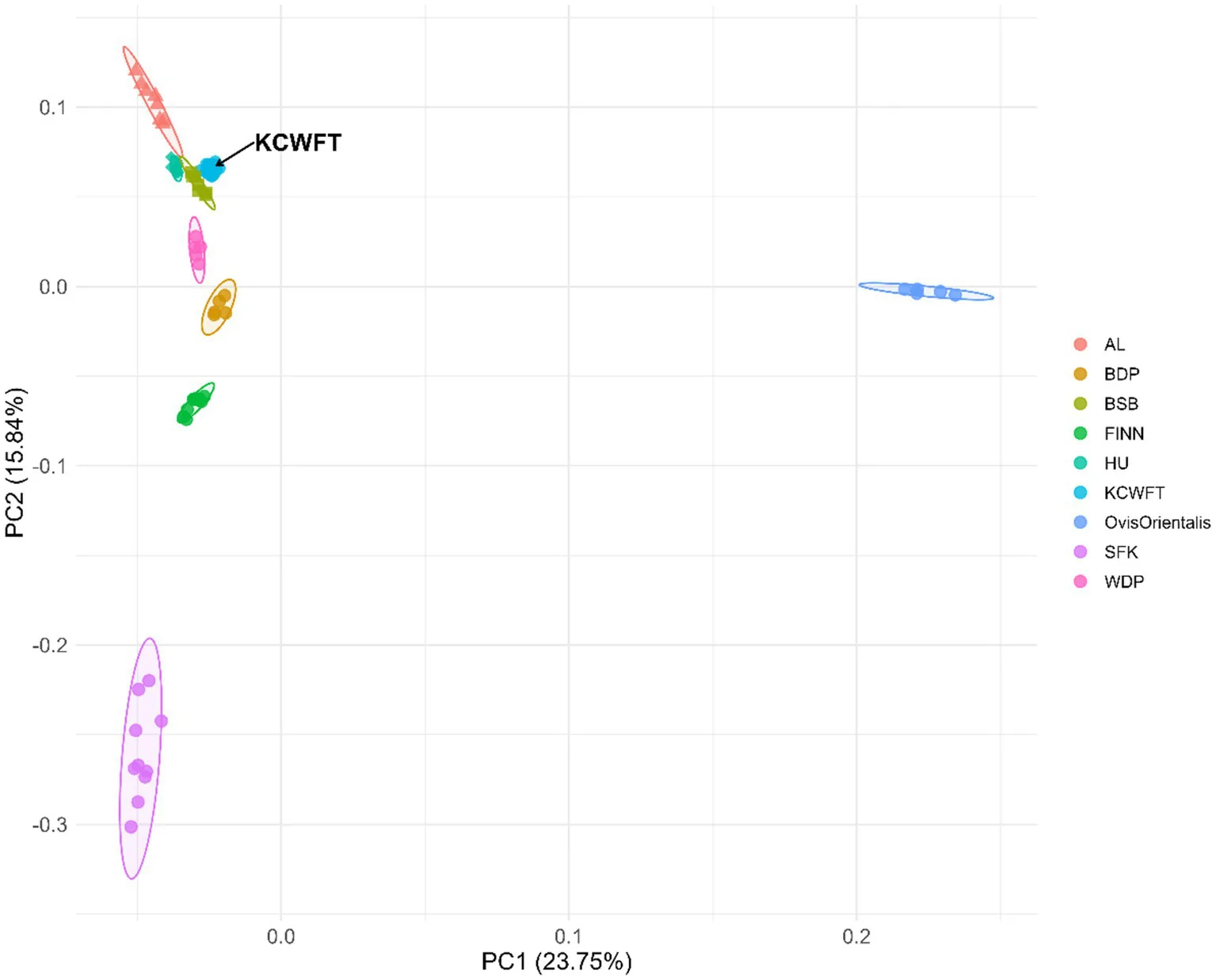
Principal component analysis (PCA) of KCWFT sheep and comparative domestic and wild sheep populations based on whole-genome sequencing data. The analysis included KCWFT (*n* = 20), six domestic sheep breeds (*n* = 62), and the wild ancestor *Ovis orientalis* (*n* = 10). Each point represents an individual animal, with colors indicating the corresponding breed or population. Ellipses illustrate the distribution of individuals within each population. *PC1* and *PC2* explain 23.75 and 15.84% of the total genetic variation, respectively. Breed abbreviations: AL, Altay; BDP, Black Dorper; BSB, Bashibai; FINN, Finnsheep; HU, Hu sheep; KCWFT, Kazakh coarse-wool fat-tailed sheep; SFK, Suffolk; WDP, White Dorper.

The Kazakh sheep also showed a clear genetic distance from the European meat (SFK) and short-tailed (FINN) breeds. The absence of dispersion within the KCWFT cluster in the plot indicates the absence of recent introgression from external lineages and the preservation of the breed genotype integrity.

Pairwise genetic differentiation estimated using the Weir and Cockerham weighted FST statistic was consistent with the PCA results. The lowest genetic differentiation was observed between KCWFT and Bashibai (FST = 0.0155), followed by Hu (0.0221) and Altay (0.0429), indicating close genetic relationships. Intermediate differentiation was detected with White Dorper (0.0581) and Finnsheep (0.0784), whereas the highest differentiation was observed with Suffolk (0.1269) and Asiatic mouflon (0.1344), reflecting their greater genetic divergence from KCWFT.

### 3.3 Selection signatures and QTL mapping

The identification of genomic regions under selection was performed using three metrics: fixation index (Fst), nucleotide diversity (*π*), and Tajima’s D statistic. Candidate selection regions were defined as windows simultaneously falling within the top 5% of the Fst distribution, the bottom 5% of the *π* distribution (reduced diversity), and the bottom 5% of the Tajima’s *D* distribution (negative values characteristic of recent positive selection). The genomic distribution of these metrics is presented in Figure 5. However, these selection signals should be interpreted with caution, as demographic history, genetic drift, recombination rate variation, and sampling effects may also contribute to extreme values of F_ST, π, and Tajima’s *D*.

**Figure 5:**
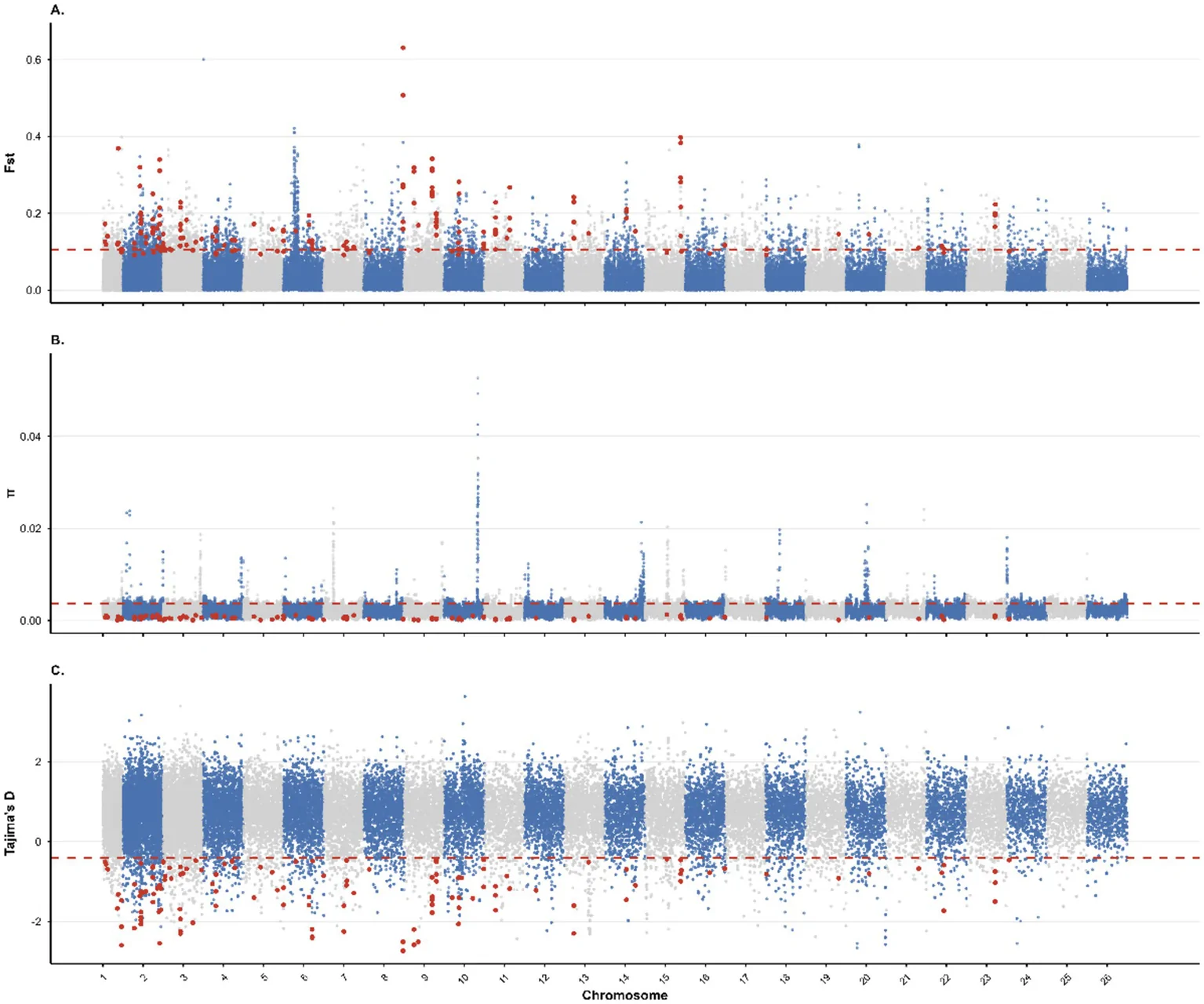
Genome-wide signatures of selection in KCWFT. Manhattan plots showing **(A)** population differentiation (*Fst*), **(B)** nucleotide diversity (*π*), and **(C)** Tajima’s *D* across 26 autosomes. Horizontal dashed lines indicate the upper 5% threshold for *Fst* and the lower 5% threshold for *π* and Tajima’s *D*. Red points represent candidate regions that simultaneously satisfy all three selection criteria (*Fst* ≥ 95th percentile, *π* ≤ 5th percentile, Tajima’s *D* ≤ 5th percentile). Chromosomes are displayed with equal width for clarity.

As a result of the integrated analysis, 208 candidate selective sweep regions were identified in the genome of the KCWFT sheep (KCWFT). Annotation of these regions using the Ensembl database (*Ovis aries* Rambouillet, release 115) identified 297 overlaps with genes. After filtering and removal of duplicates, 197 unique candidate genes were identified. Among the annotated genes, the most frequently represented were VPS13B (8 overlaps), EXOC6B (8), NF1 (7), MFSD14B (7), KATNAL1 (5), EMC2 (5), CCNY (5), and ANXA10 (5) (Supplementary Table S8). Comparison of the candidate genes with the Sheep QTLdb database (version 59) identified 44 matches corresponding to 18 unique QTL associations (Supplementary file 3). The identified QTLs were associated with health, production, and conformation traits in sheep (Table 2).

**Table 2:** QTL associations overlapping with candidate selection regions in KCWFT.

Chromosome	DA	Trait class	Candidate gene
1:171 127 562–171 127 572	Teat number	Exterior	—
2:183 107 341–183 107 351	Body weight	Production	—
3:129 877 697–129 877 707	Body weight	Production	—
3:129 877 697–129 877 707	Clinical mastitis	Health	—
3:129 877 697–129 877 707	Somatic cell score	Health	—
3:129 877 789–129 877 799	Gastrointestinal nematode resistance	Health	*SOCS2*
3:129 878 185–129 878 195	Gastrointestinal nematode resistance	Health	*SOCS2*
8:12 201 112–12 201 122	Foot rot susceptibility	Health	*CENPW*
8:12 206 868–12 206 878	Pneumonia susceptibility	Health	—
8:12 207 177–12 207 187	Pneumonia susceptibility	Health	—
10:30 787 305–30 787 315	Horn type	Exterior	—
11:18 446 880–18 446 890	Tail fat deposition	Meat	—
13:17 483 877–17 483 887	Fiber diameter	Wool	—
13:17 539 284–17 539 294	Fiber diameter	Wool	—
14:35 902 694–35 902 704	Fecal egg count	Health	—
20:30 594 708–30 594 718	*M. paratuberculosis* antibody titer	Health	—
20:30 594 708–30 594 718	*M. paratuberculosis* susceptibility	Health	—
20:30 623 273–30 623 283	*M. paratuberculosis* susceptibility	Health	—

Coordinates are based on the ARS-UI_Ramb_v3.0 assembly.

### 3.4 *PRNP* and *TMEM154* gene polymorphisms associated with genetic susceptibility to scrapie and lentiviral infections

Analysis of the *PRNP* locus identified 21 single nucleotide variants (SNVs) at 18 unique codon positions. Of these, 9 were synonymous and 12 were nonsynonymous. The amino acid substitutions included *M112T*, *G127S*, *G127V*, *A136T*, *S138N*, *N146S*, *R171Q*, *R171H*, *R171K*, *Q189L*, *T191I*, and *Q226E*.

The most frequent variant was the wild-type R171Q substitution (CGG → CAG), present in 16 animals (80.0%). Other variants at codon 171 included R171H (20%, *n* = 4) and R171K (20%, *n* = 4). Among the synonymous substitutions, the highest frequencies were observed at codons 231 and 237 (35.0% each, *n* = 7). Five haplotypes were reconstructed based on codons 136, 154, and 171 (Table 3). ARQ was the predominant haplotype with a frequency of 62.5%, followed by ARH (12.5%), ARR (12.5%), ARK (10%), and TRQ (2.5%).

**Table 3:** Distribution of PRNP genotypes and scrapie risk categories among 20 KCWFT sheep.

NSP group	Genotype	*N*	Risk of classical scrapie (Hunter, 2003)
NSP1	ARR/ARR	0	Most resistant to scrapie
NSP2	ARR/ARQ; ARR/ARH	3	Resistant to scrapie but offspring may be susceptible depending on genotype of the other parent
NSP3	ARQ/ARQ; ARH/ARH; ARQ/ARH	12	Higher risk of scrapie in these sheep and in offspring
NSP4	ARR/VRQ	0	Susceptible to scrapie but could be used as a breeding source of the ARR allele associated with resistance
NSP5	ARQ/VRQ; ARH/VRQ; AHQ/VRQ; VRQ/VRQ	0	Sheep of highest genetic susceptibility to scrapie in self and offspring
Unclassified	ARR/ARK; ARQ/ARK; ARQ/TRQ	5	
Total		20	

Genotypes were determined based on polymorphisms at codons 136, 154, and 171 of the PRNP gene. Risk classification follows the United Kingdom National Scrapie Plan (NSP) categories (80, 81).

According to the NSP classification, the ARR/ARR genotype (NSP1) was not detected. NSP2 (ARR/ARQ) included 3 animals. NSP3 (high risk), including ARQ/ARQ (*n* = 9), ARH/ARH (*n* = 1), and ARQ/ARH (*n* = 2), comprised 12 animals (60%). The remaining 5 animals (25.0%), carrying the rare ARK and TRQ alleles (genotypes ARQ/ARK, ARR/ARK, and ARQ/TRQ), were assigned to the Unclassified category, as these combinations are not included in the standard list of 15 genotypes of the NSP program.

Analysis of the key missense mutation in the *TMEM154* gene (E35K) revealed limited allelic diversity. The E35E genotype (associated with genetic susceptibility) was present in 19 of 20 animals (95.0%), including 19 homozygotes (G/G) and one heterozygote (A/G). The protective K35K genotype was not observed. One animal (sample ID 4) carried the heterozygous K35E genotype, indicating one copy of the protective haplotype 1 (K35) and one copy of the susceptible haplotype (E35).

## 4 Discussion

The present study represents the first comprehensive whole-genome sequencing (WGS) analysis of the KCWFT sheep, an indigenous breed of Kazakhstan representing an important genetic resource. Using high-coverage WGS data from 20 individuals and integrating them with publicly available genomes of six domestic breeds and the wild ancestor *Ovis orientalis*, we characterized the population structure, identified genomic signatures of selection, and assessed the genetic risk profile for two major infectious diseases. These results provide a fundamental genomic basis for understanding the evolutionary history and adaptive potential of this historically important breed. One of the limitations of this study is the relatively small sample size (*n* = 20). Although this is sufficient for the first genome-wide characterization of the KCWFT sheep breed and the identification of key genetic features, this sample size limits the ability to detect rare genetic variants and reduces the accuracy of estimating the frequencies of rare alleles. Therefore, the obtained results require further validation in larger and more geographically diverse samples.

### 4.1 Population structure

Principal component analysis (PCA) based on a set of LD-independent SNPs revealed clear clustering of the Kazakh sheep (KCWFT) into a single group that was genetically close to the Asian fat-tailed breeds Bashibai (BSB), Hu (HU), and Altay (AL), but distant from the European breeds (SFK, FINN). These results are consistent with the hypothesis of historical genetic continuity and gene flow among sheep lineages of Western, Central, and Eastern Asia (10). Separation along PC1 captured the domestication signal, distinguishing *O. orientalis* from all domestic sheep, thereby supporting the theory that the species originated in the Fertile Crescent approximately 10,000–11,000 years ago (10, 12). Meanwhile, PC2 reflected the geographical patterns of breed formation, separating Asian and European lineages, which is consistent with the important role of geographic isolation as a driver of genetic differentiation in sheep. The genetic proximity of KCWFT to the Bashibai and Altay breeds is likely explained by their formation within the same transboundary region of Kazakhstan and Xinjiang, where historical seasonal migration routes promoted long-term gene flow among populations (10). Similarly, the proximity of KCWFT to the Hu breed is consistent with whole-genome modeling data indicating that this breed originated from ancient populations of Northern China and Central Asia that diverged approximately 5-6 thousand years ago (79). The observed clustering is consistent with the existence of a common Asian genetic background of indigenous breeds that spread along historical migration routes, including the Great Silk Road (4, 8, 10). Recent SNP-chip studies have demonstrated that KCWFT sheep exhibit high genetic diversity and form a distinct phylogenetic cluster that differs from both East Asian and European populations (2, 4). The obtained results are consistent with previous studies and extend them by providing higher-resolution data on the within-breed genetic architecture of KCWFT. The compact distribution of KCWFT samples on the PCA plot indicates high genetic homogeneity of the breed and reveals no evidence of pronounced recent introgression, which is of great importance for genetic resource conservation programs.

Despite the overall isolation of the KCWFT cluster by the phylogenetic tree, its position on the tree shows the closest phylogenetic proximity to the Altay sheep, which may reflect a common origin or historical genetic links between these breeds (35). However, a distinct separation remains between the KCWFT and Altay sheep clusters, confirming the independence of the KCWFT breed (35). It should be noted that within the KCWFT cluster, most animals are characterized by very short internal branches, indicating a relatively low level of genetic differentiation between individuals and a high level of genetic consolidation within the studied population. All samples are reliably grouped within a single branch without mixing with other breeds, confirming the correctness of the breed identification and the absence of signs of recent introgression in the sample studied. Unlike the KCWFT, some other breeds, such as Finnish sheep, Suffolk, and Asiatic Mouflon, are characterized by longer internal branches and greater structure, reflecting higher intrabreed genetic diversity. At the same time, White Dorper, Black Dorper, Hu sheep, and Bashibai also form their own distinct clusters without overlapping with the KCWFT. Thus, the phylogenetic tree confirms that the KCWFT breed represents a genetically distinct and well-consolidated population, distinguished by high intrabreed homogeneity (4). The closest position to the Altay sheep is consistent with the geographic distribution and probable historical connections of Central Asian breeds, but the clear separation of the clusters indicates the formation of the KCWFT as a distinct genetic lineage (35). The obtained results confirm the uniqueness of the KCWFT gene pool and emphasize the importance of preserving this indigenous breed as a valuable genetic resource for Kazakhstan.

### 4.2 Genomic signatures of selection and adaptive evolution

The integrated analysis of three selection metrics (Fst, *π*, and Tajima’s *D*) identified 208 candidate selective sweep regions containing 197 unique candidate genes (Supplementary file 3). These findings suggest that both natural and artificial selection have contributed to shaping the genome of the KCWFT sheep. However, these selection signals should be interpreted with caution, as demographic history, genetic drift, recombination rate variation, and sampling effects may also contribute to extreme values of F_ST, *π*, and Tajima’s *D*. A pronounced nucleotide diversity peak was observed on chromosome 10 (70.7–72.2 Mb). Annotation of this interval identified 11 loci, including a tandem cluster of five highly similar ABCC4-like paralogs spanning approximately 1.2 Mb, together with several protein-coding, non-coding, and pseudogene loci. The elevated nucleotide diversity in this interval may therefore be related to the complex duplicated structure of this genomic region; however, further investigation is required to determine the biological significance of this signal. The most frequently occurring genes within the selection regions were *VPS13B*, *EXOC6B*, *NF1*, *MFSD14B*, *KATNAL1*, *EMC2*, *CCNY*, *ANXA10*, *TBXT*, *PIP4K2B*, *OMG*, *CHAF1B*, *CCDC93*, *ANKRD10*, and *ANAPC2*. Of particular interest are *SOCS2*, *TBXT*, *BTN3A3*, *BTN1A1*, *ADAMTS5*, *ALCAM*, and *CD247*, which have previously been associated with the regulation of growth, tissue development, immune response, and production traits (36–38).

Among the candidate genes, VPS13B was distinguished by the highest number of overlapping selection windows (*n* = 8), unequivocally indicating an extended, highly differentiated selection region (35). This finding is not unexpected, as the VPS13 (vacuolar protein sorting 13) family has consistently been reported in studies of adaptation in ruminants. Its homologs have been identified under selection in Mediterranean and Chinese sheep, Moroccan and Mediterranean goats (39, 40), as well as in pigs from southern China in the context of heat adaptation (41). In a recent study of Mediterranean breeds (40), VPS13B was identified within heterozygosity-rich regions (HRRs) associated with climatic adaptation in fat-tailed Barbarine sheep (40). The authors also noted that the VPS13B protein is involved in adipocyte formation and development (42), which is particularly relevant for fat-tailed breeds in which adipocytes accumulate in the tail fat depot. An additional functional role of VPS13B is also noteworthy: according to QTL mapping in cattle and buffalo databases, the gene was detected within a region associated with limb length, which is closely related to fertility and milk production (40). Notably, an evolutionary analysis of introgression in domestic sheep identified VPS13B as the gene showing the strongest introgression signal from the wild urial mouflon (43), highlighting its critical importance in the formation of domestic breeds. For KCWFT, these findings collectively suggest that selection on VPS13B may have simultaneously enhanced the adipogenic potential of the fat tail, climatic resilience to the sharply continental conditions of Kazakhstan, and the biomechanics of the musculoskeletal system required for long-distance seasonal migrations under transhumant livestock management (35).

The TBXT locus (freq. 3) is a key regulator of mesoderm development, whose role in determining tail length has been confirmed directly in Kazakh breeds (44, 45). The c.334G>T (Gly112Trp) mutation in the T-box domain, in linkage with the c.333G>C variant, is associated with the tailless phenotype in fat-tailed sheep, which is consistent with the KCWFT phenotype (38, 44). The recessive CT/CT genotype causes shortening of the caudal vertebrae, as confirmed by crossbreeding experiments. Studies conducted in 2025 detected the accumulation of the Brachyury protein in the embryonic tail bud, confirming its direct role in tail segmentation (44, 46).

The EXOC6B gene (freq. 7) encodes a subunit of the complex required for exocytosis and cell polarity (47). Of particular importance is the association of EXOC6B with the secretory function of pancreatic β-cells (45, 47). For fat-tailed sheep, whose metabolism depends on the efficiency of the insulin response during the grazing season, selection on EXOC6B may reflect adaptation of mechanisms involved in fat accumulation and survival during seasonal feed deprivation.

Selection signal at the KATNAL1 locus (freq. 4) highlights the importance of the genetic regulation of fertility in Kazakh sheep. This gene encodes a subunit of the microtubule-severing enzyme katanin, which is critically required for meiotic spindle assembly and spermatid morphogenesis (48). Studies have confirmed that KATNAL1 performs indispensable functions in sperm flagellum formation, manchette regulation, and spermiation (the release of spermatozoa from the epithelium), which likely plays a role in the fertility of the breed (48, 49). In addition, the RNF212 gene, which stabilizes crossover protectors during meiosis, was identified within this region (50). In sheep, this locus explains up to 46% of the heritable variation in recombination rate, which likely makes selection on RNF212 adaptively important for maintaining genetic diversity and efficient reproduction in KCWFT populations (51).

Several additional candidate genes may also have contributed to the adaptation of KCWFT sheep. SOCS2 is a key regulator of the JAK/STAT signaling pathway and growth hormone signaling, suggesting that selection at this locus may influence postnatal growth and productive performance under extensive grazing conditions (36, 52). NF1 participates in Ras-mediated signaling involved in cell proliferation and immune regulation, indicating a potential role in physiological adaptation and resilience (53). BTN3A3, a member of the butyrophilin family, is involved in the activation of γδ T cells, which are abundant in ruminants and play an important role in innate immune responses (54). Selection acting on these loci may therefore reflect the combined effects of natural adaptation to harsh environmental conditions and artificial selection for productive and adaptive traits in the.

### 4.3 Immunity, innate defense, and resistance to parasites

The most significant selection signal was identified in the chromosome 3 (OAR3) region, where the SOCS2 (*suppressor of cytokine signaling 2*) gene is located. This locus overlaps with three independent QTLs in the Sheep QTLdb database: resistance to nematodes (FEC), clinical mastitis, and milk somatic cell count. The SOCS2 protein acts as a negative regulator of the JAK/STAT pathway, modulating the balance of T-helper cells and the level of IL-3, which is required for the initiation of protective mastocytosis during parasitic invasion (36, 37). The high significance of this locus is supported by its identification among the seven most differentiated candidate genes (together with C3AR1, CSF3, NOS2, and others) in Fst outlier analyses of sheep infected with *H. contortus* (14). The presence of this signal in the Kazakh breed is biologically consistent with its evolution under year-round grazing conditions with a high seasonal nematode burden, where survival was ensured by genetically determined immunocompetence. It is important to consider the pleiotropic effect of this locus: mutations in *SOCS2* are associated not only with immunity but also with increased body weight and body size. Thus, the *SOCS2* region in the genome of Kazakh sheep is likely the result of the combined action of natural selection for survival and artificial selection for meat productivity. Another significant pattern of immune selection is represented by the *BTN3A3* and *BTN1A1* gene cluster. Butyrophilins are key regulators of the adaptive immune response through interaction with γδ T lymphocytes, which, unlike in humans, represent the predominant circulating T-cell population in sheep (55). The ability of butyrophilins to form functional heteromers (e.g., *BTN2A1/BTN3A1*), discovered in 2024, provides additional significance to the simultaneous presence of several genes of this family within the KCWFT selection regions (56). The *BTN3A3* gene has been identified as a critical protective factor against influenza A viruses (including H5N1): it blocks viral replication at early stages by specifically targeting the viral nucleoprotein (57, 58). Although this function has been demonstrated primarily in humans and its conservation in sheep has not yet been established, these findings indicate the multifunctional nature of butyrophilin family proteins, combining immunoregulatory and antiviral properties. Accordingly, the detection of *BTN3A3* and *BTN1A1* within candidate selection regions may reflect selection acting on mechanisms of innate and adaptive immunity that contribute to increased overall resistance of animals to infectious agents. Given that the functional role of butyrophilins in sheep remains poorly understood, the selection signal identified in this study is of considerable interest and deserves further functional validation (56). The *ALCAM* and *CD247* genes also belong to the group of genes with immunological significance (59, 60). The importance of *ALCAM* is supported by its identification in adaptation regions of sheep from diverse environments ranging from the tropics to the Arctic, indicating its role as a universal marker of adaptation to extreme conditions (59). Considering the ability of helminths to suppress ζ-chain expression in order to evade host immune surveillance, the detection of a selection signal at the *CD247* locus in Kazakh sheep likely reflects an evolutionary adaptation aimed at maintaining the functional competence of T cells under conditions of chronic pasture parasitism (60). The *SIGLEC1* (*sialoadhesin*, CD169) gene, showing a selection signal in Kazakh sheep (KCWFT), was previously considered a specific marker of myeloid lineage cells (macrophages and CD14 + monocytes), mediating the recognition and internalization of sialylated pathogens, including retroviruses and *Campylobacter jejuni* (61, 62). However, in sheep and other ruminants, the *SIGLEC1* protein has also been detected in the epithelium of the male reproductive tract and on spermatozoa, indicating its possible involvement in the regulation of reproductive processes (63). In addition, *SIGLEC1* expression is induced by type I interferons and is markedly increased during viral infection and inflammation, reflecting its important role in the activation of innate immunity (61).

### 4.4 Adaptation to extreme environmental conditions and regulation of energy metabolism

The results of the population genetic analysis confirm that the Kazakh sheep group (KAZ) belongs to the populations whose genomic history has been shaped by adaptation to extreme environments (4, 35). The NF1 gene was previously proposed as a candidate for adaptation to high-altitude conditions because of its role in the regulation of angiogenesis; however, more recent studies have shown that the *NF1, EVI2A, EVI2B,* and *OMG* gene complex is also associated with the adaptive response to physical exhaustion and fat deposition in sheep (8, 35, 64). Considering the origin of KCWFT under the arid pasture conditions of Kazakhstan, where animals experience prolonged seasonal migrations, limited feed resources, and pronounced temperature fluctuations, the detected selection signal most likely reflects complex adaptation to prolonged physical exertion and the characteristics of energy metabolism (2, 4, 6). At the same time, it cannot be excluded that the high frequency of NF1 detection is partially attributable to within this chromosomal block. The PYGM gene (muscle glycogen phosphorylase), identified within the KCWFT selection region, plays a key role in energy metabolism by catalyzing glycogen breakdown in skeletal muscle for rapid ATP production (65). The biological significance of this locus is supported by a natural model of its deficiency in sheep from Western Australia: a mutation at the splice site of intron 19 results in the development of McArdle disease (glycogen storage disease), manifested by exercise intolerance (65, 66). The importance of this mechanism is further supported by recent studies in Bayinbuluk sheep, which demonstrated that enhanced glycolytic flux in skeletal muscle represents a fundamental response to high-altitude hypoxia (67). Thus, positive selection at the PYGM locus in Kazakh sheep likely conferred a survival advantage by enabling them to maintain high muscular activity under conditions of seasonal migrations and limited resources (65).

### 4.5 *PRNP* polymorphisms: assessment of resistance

Analysis of the prion protein (*PRNP*) gene in the studied KCWFT population revealed a genotype distribution indicating moderate genetic susceptibility to classical scrapie. The predominance of the ancestral ARQ haplotype (62.5%), the absence of the highly resistant ARR/ARR genotype (NSP1), and the predominance of the NSP3 category (60%) indicate the preservation of a genetic profile characteristic of populations that have benot undergone intensive selection for disease resistance (24, 68). These results are consistent with the current understanding that the ARQ haplotype represents the ancestral form of the *PRNP* gene in domestic sheep (*Ovis aries*), from which the remaining variants originated during the course of evolution and selection (17, 69). Despite the limited sample size (n = 20), the observed distribution of *PRNP* alleles and genotypes generally corresponds to published data for other indigenous sheep breeds (24, 70). For example, the frequency of ARQ in the Turkish Zom breed reaches 65.2%, whereas the ARR/ARR genotype occurs in only 6.52% of animals, while in the Chinese Hu breed its frequency is approximately 1.67% (24). In contrast, in European breeds, such as the Portuguese Serra da Estrela, both the ARR allele (58.7%) and the ARR/ARR genotype (18.5%) are substantially more frequent as a result of long-term selection for scrapie resistance (17). Thus, despite the small sample size, the observed patterns are consistent with published data from larger populations and reflect the absence of systematic scrapie control programs in the studied breeds (68). The absence of the VRQ haplotype, which is associated with high genetic susceptibility to scrapie, is consistent with data from other indigenous Asian breeds, in which it is also extremely rare or absent, unlike in some European populations (24). A notable feature of the studied population was the identification of rare PRNP genotypes not included in the standard international classification (ARR/ARK, *n* = 2; ARQ/ARK, *n* = 2; ARQ/TRQ, *n* = 1), which were detected in 25% of the animals. Despite the limited sample size, the presence of these variants indicates the preservation of high genetic diversity of the *PRNP* gene, characteristic of indigenous sheep populations (24, 71). Of particular interest is the ARK allele, identified in five animals (allele frequency 25%). In most studied sheep populations worldwide, this variant occurs at a much lower frequency: 0.35% in the Turkish Kıvırcık breed (71), approximately 1.1% in the Portuguese Serra da Estrela breed (17), and ranging from 0.3 to 4.1% in the Israeli Assaf and Awassi breeds (70). Experimental studies have shown that the K171 allele is associated with increased resistance to classical scrapie: KK171 homozygotes exhibit high resistance to infection, whereas QK171 heterozygotes are characterized by an extended incubation period of the disease. Therefore, the detection of ARR/ARK carriers in the studied population may indicate the presence of additional protective PRNP variants that are not considered in the standard NSP classification system (33, 71). The TRQ allele was identified in one animal (allele frequency 2.5%). This variant is considered rare and occurs predominantly in indigenous breeds from the Mediterranean and the Middle East (24, 39, 71). Similar frequencies have been reported for the Turkish Zom breed (6.5%), the Chios breed (4.44%), and Greek sheep (approximately 1.6%) (24, 71). The detection of TRQ in the studied KCWFT population may reflect the preservation of ancient PRNP variants characteristic of populations that originated near the historical centers of sheep domestication. In addition to the classical polymorphisms, PRNP sequencing identified 21 single nucleotide variants across 18 codons, including 12 nonsynonymous substitutions—M112T, G127S, G127V, A136T, S138N, N146S, R171Q, R171H, R171K, Q189L, T191I, and Q226E—indicating a high level of allelic diversity in the studied population tur (71–73). The Q226E variant has previously been described in wild ruminants, particularly in red deer (*Cervus elaphus*) (74). Its identification in KCWFT sheep complements current knowledge of PRNP variability in indigenous sheep breeds. The identified M112T and N146S substitutions deserve particular attention: M112T has been associated with resistance to BSE and scrapie in British sheep, whereas N146S has a proven protective effect in goats (18, 24, 73). It has been suggested that this substitution may play a similar protective role in sheep, making it a promising marker for breeding programs. The presence of G127S and G127V substitutions at codon 127 has previously been reported in other indigenous breeds from Turkey, Ethiopia, and Indonesia (68, 71, 73, 75). Codon 127 is part of the highly conserved glycine-rich motif (GAVVG126G127LGGYMLG), which plays an important role in preventing amyloid fibril formation, thereby inhibiting the development of prion diseases (68, 75). The identified A136T, S138N, and Q189L substitutions are consistent with data reported for indigenous breeds from Asia and the Mediterranean. In particular, the Q189L variant is considered an indicator of the Asian origin of sheep (72, 73). Its identification in KCWFT complements the PCA results demonstrating the genetic proximity of this breed to other Asian fat-tailed sheep (70, 73). Taken together, these results indicate that the PRNP gene in the studied KCWFT population is characterized by a combination of widespread ancestral haplotypes and several rare regional variants. The results, based on a sample of 20 animals representative of a local flock, require confirmation in a larger sample before definitive conclusions can be drawn regarding the genetic status of the breed as a whole. Nevertheless, the absence of the ARR/ARR genotype among the studied animals and the predominance of susceptible genotypes suggest that the frequency of resistant alleles in the KCWFT population may be low. If this trend is confirmed in a larger sample, breeding programs aimed exclusively at increasing the frequency of the ARR allele should be implemented with caution, as they may reduce the unique genetic diversity and adaptive potential of KCWFT. Accordingly, promising directions for future research include expanding the sample size to refine *PRNP* allele frequencies, functional evaluation of the rare ARK and TRQ variants, and monitoring the current epizootic status of scrapie in Kazakhstan. These measures are necessary to move from passive surveillance to an active genetic risk management system, thereby ensuring the country’s long-term veterinary security.

### 4.6 *TMEM154* polymorphism and genetic susceptibility to Maedi-Visna

Analysis of the key missense mutation in the *TMEM154* gene (E35K, rs408593969) revealed a low frequency of the protective K35 allele in the studied KCWFT population. The frequency of the protective K35 allele was only 2.5% (1 allele out of 40). Nearly complete fixation of the E35 allele, associated with increased genetic susceptibility to SRLV, was observed, with 95.0% of the animals (*n* = 19) being EE35 homozygotes. The observed frequency of the protective K35 variant (2.5%) in the KCWFT population shows a clear difference from that reported for most sheep populations worldwide (76). In particular, it is substantially lower than in the North American Rambouillet and Suffolk breeds (20). Similarly pronounced differences are observed when compared with recent studies: the K35 frequency in Kazakh sheep is an order of magnitude lower than that reported for the Navajo-Churro, Barbados Blackbelly, and even the Brazilian Santa Inês population, which is considered relatively susceptible (22, 76–78). Such a pronounced depletion of the protective haplotype 1 indicates that the studied population may be characterized by increased genetic susceptibility in the event of virus introduction. According to published studies, carriers of the E35 allele have a substantially higher risk of SRLV infection than K35K homozygotes (20, 76). Thus, the obtained results indicate a low frequency of the protective K35 allele in the studied KCWFT population and emphasize the absence of directed selection at the TMEM154 locus during the formation of this breed. Since the conclusions are based on a limited sample size (*n* = 20), they require confirmation using a more representative dataset including animals from different flocks and regions of Kazakhstan. If the observed trend is confirmed, expanded genetic screening of sheep populations will allow an objective assessment of the distribution of the protective K35 allele and determine the feasibility of its use in breeding programs aimed at increasing resistance to lentiviral infections while preserving the genetic diversity of indigenous breeds.

However, these findings should be interpreted as genetic predisposition rather than confirmed disease susceptibility, as the present study was based on genotype frequencies and did not include phenotypic, clinical, or epidemiological validation.

From a practical perspective, the genomic information generated in this study provides a foundation for the development of breeding and conservation strategies for KCWFT sheep in Kazakhstan. Candidate variants and selection signatures associated with adaptation, production, and disease-related traits could be further validated and incorporated into genomic selection programs, while monitoring genetic diversity could help prevent excessive loss of locally adapted genetic resources. Following validation in larger populations, *PRNP* and *TMEM154* genotyping could complement veterinary surveillance and breeding programs by identifying animals carrying alleles previously associated with increased or reduced genetic susceptibility.

The present study has several limitations that should be considered when interpreting the disease-associated genetic findings. The analysis included only 20 animals from a single farm; therefore, the observed *PRNP* and *TMEM154* allele and genotype frequencies should not be considered representative of the entire KCWFT population in Kazakhstan. In addition, clinical, serological, and epidemiological data, including herd history, animal movements, inter-herd contacts, and infection status, were not available. Consequently, the identified genotypes indicate genetic predisposition rather than confirmed disease susceptibility or epidemiological risk. In particular, the high frequency of the E35 allele and the absence of the KK35 genotype should be validated in larger and geographically diverse KCWFT populations, ideally in combination with confirmed infection-status data.

## 5 Conclusion

This study represents the first comprehensive analysis of whole-genome data on the KCWFT sheep breed using WGS technology. The results allowed us to characterize the population’s genetic structure, identify selective regions of the genome associated with adaptation and economically valuable traits, and assess the animals’ genetic predisposition to two important infectious diseases. Analysis of the population structure and phylogenetic relationships revealed that the KCWFT is a genetically distinct and well-consolidated breed, most closely related to the Altai sheep breed, while maintaining distinct genetic distinctiveness. High within-breed homogeneity and the absence of signs of recent introgression confirm the preservation of the unique gene pool of this indigenous breed. A comprehensive search for selective regions identified 197 candidate genes involved in the regulation of growth, fat-tail development, reproductive function, immunity, energy metabolism, and adaptation to extreme environmental conditions. Of particular interest are the *VPS13B*, *SOCS2*, *TBXT*, *EXOC6B*, *KATNAL1*, *BTN3A3*, *BTN1A1*, *PYGM*, *NF1*, and other genes, which likely played a key role in shaping the breed’s adaptive characteristics in Kazakhstan’s harsh continental climate and year-round grazing conditions. Analysis of *PRNP* and *TMEM154* gene polymorphisms revealed that the studied population is characterized by a predominance of genotypes associated with susceptibility to classical scrapie and Maedi-Visna, along with a number of rare *PRNP* variants that are of interest for further research. The obtained results highlight the need for expanded genetic monitoring of Kazakh sheep populations and the development of breeding programs aimed at increasing resistance to infectious diseases while preserving genetic diversity. Thus, this work significantly expands our current understanding of the genetic architecture of the KCWFT sheep breed and forms the basis for further research into the evolution, adaptation, and genetic improvement of local breeds. The obtained genome-wide data programs and the scientific and practical value for preserving Kazakhstan’s genetic resources, developing genomic selection programs, and improving the efficiency of sustainable sheep farming in Central Asia.

## Data Availability

The datasets generated during this study are available through the NCBI BioProject repository under accession PRJNA1494197. The whole-genome sequencing data for the 20 samples are available in the NCBI Sequence Read Archive (SRA) under the following accession numbers: SRR39821617–SRR39821636.
